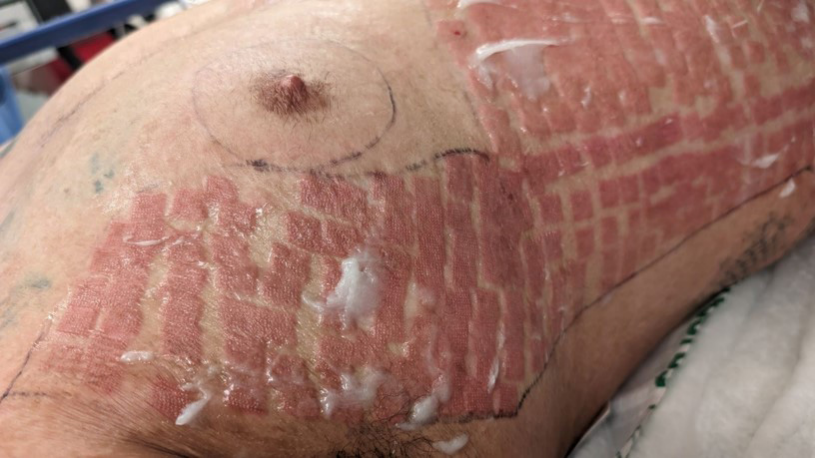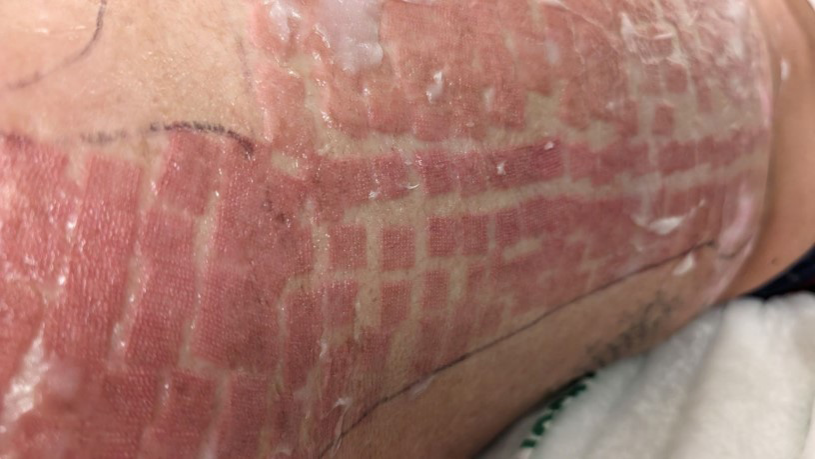# 930 Laser-Induced Urticaria in a Burn Patient with Hypertrophic Scarring: A Case Report

**DOI:** 10.1093/jbcr/iraf019.461

**Published:** 2025-04-01

**Authors:** Maysa Shemmiyeva, Namratha Mohan, Adel Aziz, Alan Pang

**Affiliations:** Texas Tech University Health Sciences Center School of Medicine; Texas Tech University Health Sciences Center School of Medicine; Texas Tech University; Texas Tech University

## Abstract

**Introduction:**

Urticaria is vascular skin reaction characterized by transient, pruritic, erythematous wheals. While often idiopathic, urticaria can be triggered by various stimuli including foods, medications, infections, physical factors, and external triggers.

Laser-induced urticaria: rare form of physical urticaria in which wheals develop following exposure of the skin to laser light. Pathogenic mechanisms underlying laser-induced urticaria are not yet fully understood. May involve laser-induced thermal injury, generation of novel antigens from altered skin proteins, direct mast cell activation, or production of reactive oxygen species. Unlike other physical urticarias (e.g., those caused by heat or cold), laser-induced urticaria often does not respond well to conventional treatments (e.g., antihistamines).

There is currently an absence of literature discussing laser-induced urticaria following treatment for pruritic grafting burn scar hypertrophy. We present a case involving a 33-year-old male who developed pruritic hypertrophic scarring after multiple grafting procedures and underwent laser treatment to alleviate symptoms, resulting in laser-induced urticaria that resolved spontaneously within 7 days without airway compromise.

**Methods:**

This study is designed as a detailed case report, documenting the incidence, treatment, and outcomes of laser induced urticaria in a single patient. Study phases: initial incident, acute treatment and post-acute phase, scar management phase, complications and results.

**Results:**

The lack of additional treatment beyond discontinuation of laser therapy and post-operative Benadryl administration suggest that the reaction was self-limiting. Spontaneous resolution of the laser-induced urticaria may support the theory that laser-induced urticaria is a localized, temporary hypersensitivity reaction rather than a more generalized immune/allergic response. Absence of airway compromise or more severe systemic complications suggests while laser-induced urticaria may cause discomfort, it may not pose risk for severe complication as other physical urticarias, though further study is needed.

Further research should explore how scar tissue characteristics or grafted areas may contribute to the development of laser-induced urticaria in burn patients

**Conclusions:**

This case offers a unique clinical presentation of laser-induced urticaria that contributes to existing limited literature. Findings of this case underscore the need for further research on pathophysiological mechanisms of laser-induced urticaria. Laser-induced urticaria, though rare, should be considered as a potential adverse reaction, particularly in the context of grafting pruritic scar treatment.

**Applicability of Research to Practice:**

Our case report showed that laser induced urticaria is a temporary hypersensitivity reaction with local presentation and can be managed with supportive care and Benadryl

**Funding for the Study:**

N/A